# A BWM-TOPSIS Hazardous Waste Inventory Safety Risk Evaluation

**DOI:** 10.3390/ijerph17165765

**Published:** 2020-08-10

**Authors:** Fumin Deng, Yanjie Li, Huirong Lin, Jinrui Miao, Xuedong Liang

**Affiliations:** 1 Business School, Sichuan University, Chengdu 610065, China; dengfm@scu.edu.cn (F.D.); liyanjie@stu.scu.edu.cn (Y.L.); 2018225025102@stu.scu.edu.cn (J.M.); 2 The Economy and Enterprise Development Institute, Sichuan University, Chengdu 610065, China; 3 National Environmental Protection Hazardous Waste Disposal Engineering Technology (Chongqing) Center, Chongqing 401120, China; linhuirong@zthbjt.com

**Keywords:** hazardous waste, sustainable development, inventory management, risk evaluation, BWM, TOPSIS

## Abstract

Hazardous waste can cause severe environmental pollution if not disposed of properly, which in turn can seriously affect the sustainable development of the entire ecology and will inevitably bring disaster to companies. However, because of limited available disposal capacity, it is often difficult to safely dispose of hazardous waste, meaning that it must be kept as passive inventory. For the passive inventory of hazardous waste, risk evaluation of safe operation of the inventory is crucial and urgently needs to be resolved. Based on this, this paper focuses on the risk management of hazardous waste inventory of waste-producing companies and proposes a risk evaluation system for safely dealing with hazardous waste inventory, which expands the scope of inventory safety management and provides guidance to companies on developing appropriate measures to ensure hazardous waste inventory safety. First, the risk evaluation index system for hazardous waste inventory is constructed from equipment, management level, nature of hazardous waste and operational aspects. Then, the best worst method (BWM) is employed to calculate the criteria weights and the technique for order performance by similarity to ideal solution (TOPSIS) is employed to rank the alternatives. Finally, risk evaluation on four waste-producing companies was conducted using the developed method. The results show that Case Company 4 has the greatest risk of hazardous waste inventory, which should be reduced by improving storage method and the amount of hazardous waste. It was found that the proposed evaluation system was effective for hazardous waste inventory safety risk assessments and that the designed index system could assist companies improve their hazardous waste inventory management.

## 1. Introduction

In recent years, the hazardous waste output has remained high in many countries and most of them have increased. This paper uses the hazardous waste output of various countries of the United Nations Statistics Division (some countries have data every other year) as cross-sectional data and the time series from 2010 to 2017 as a time series. The panel data of the hazardous waste output of some countries in the world are shown in Table 1 (the unit is tonne).

Further, with the revisions in government laws and regulations and improvements in people’s awareness of environmental protection, hazardous waste management has become more important. As hazardous waste is solid or liquid waste that is corrosive, toxic, flammable, reactive or infectious [1], if an accident occurs in the logistics process, it could cause huge casualties and property damage, endanger the environment and soil and cause social panic. On 12 August 2015, an explosion occurred in the hazardous material warehouse of Ruihai Company in Binhai New Area, Tianjin, China, killing 165 people, with 8 missing people, and injured 798 people. The rating agency Fitch warned that insured losses of the Tianjin explosion may be up to $1.5 billion [2]. Considering the huge dangers of hazardous material, strengthening the risk management of hazardous waste warehouses is an issue that urgently needs attention in related industries.

In the relevant literature on hazardous waste management, most hazardous waste management research has been focused on hazardous waste route planning [3,4,5,6,7] and disposal [8,9,10,11,12], solving only the back-end safe transportation and disposal process, while the problem of the source in waste-producing companies is not dealt with. There are few studies on safety management of hazardous waste inventory. In fact, the passive inventory of hazardous waste has become an indispensable part of hazardous waste management. In China, for example, the National Bureau of Statistics reported that, in 2017, the hazardous waste output was 69.37 million tonnes but the hazardous waste disposal was only 25.52 million tonnes; the disposal capacity of hazardous waste is far behind the amount of hazardous waste generated. Therefore, most waste-producing companies have large hazardous waste inventories, which is a significant safety hazard and focused risk evaluation procedures are required, which means that the development of hazardous waste inventory safety risk evaluation is significant for effective hazardous waste inventory management. 

To help companies reduce the risk of hazardous waste inventory and improve the level of inventory management of hazardous waste, a waste-producing companies hazardous waste inventory risk evaluation index system is developed focused on the four aspects of equipment, management level, the nature of the hazardous waste and operations. Then, a BWM-TOPSIS hybrid method is employed to evaluate the safety risks associated with hazardous waste inventory. Finally, a case study is given to prove the viability of the proposed feasibility of hazardous waste evaluation index system and provide a reference for hazardous waste inventory management. 

### 1.1. Inventory Management

At present, in the field of hazardous waste management, there are few articles discussing the safety risk evaluation of the inventory of hazardous waste in individual companies. Hazardous waste inventory risk assessment is part of inventory management. However, there is a significant difference as the objects in general inventory are valuable, whereas hazardous waste is worthless but cannot be discarded at will; therefore, it is necessary to ensure proper hazardous waste industry management.

Duong et al. recommended using multi-metric approaches such as order rate variance ratio, average inventory, and fill rate for perishable product inventory management, and proposed appropriate performance metrics to evaluate the entire system and provide robust solutions [13]. Gu et al. proposed two non-linear programming models for aircraft parts inventory management that predict impending demand based on the fault distribution of the installed parts and minimize the total costs by determining optimal order times and order quantities [14]. Najafi et al. claimed that the challenge for the inventory management of blood, which is a perishable product, was to hold enough inventory to ensure a high supply level while minimizing maturity losses [15]. Saha and Ray concluded that healthcare system inventory management needed to be compatible with its operations and key features to ensure that the inventory-related costs were minimized, service levels maximized and treatment prices and resource waste reduced [16].

Most inventory management research has focused on minimizing total costs, improving service levels, reducing total costs by determining optimal order times and order quantities and holding sufficient inventory to increase supply levels; however, to date, there has been little research on waste inventory management. Cho et al. developed a new probabilistic safety assessment method to deal with the safety assessment uncertainties associated with high-level radioactive waste repositories, in which the waste repository risk is presented as a product of the failure probability density of the repository and the radionuclide inventory in the repository when a repository failure occurs [17]. Louis and Shih established a dynamic inventory model to minimize the cost of urban domestic waste recycling systems by establishing a recyclable waste inventory warehouse and using historical s-hand material prices as the external input [18]. Mes et al. studied the inventory routing of dynamic waste collection, with a particular focus on the collection of waste in underground containers, in which the waste “inventory” was emptied rather than replenished, which assisted waste collection companies in planning when to empty the containers and how to arrange the vehicle routes to minimize collection costs and maximize customer satisfaction [19]. Zhang et al. developed a multi-level, multi-cycle solid waste management system (MSWM) and claimed that, as product demand in multi-level management of traditional supply chains is a random variable that varies with distribution and the MSWM waste generation rate is random, assuming an average inventory level could provide decision support for real-time and long-term municipal solid waste planning [20]. Zhao and Ke incorporated inventory risk into an optimized location routing model for explosive waste, and then conducted a comprehensive explosion risk assessment to minimize overall system costs and environmental risks [21].

The research object of this paper is different from the traditional inventory management of raw materials. Traditional inventory management mainly studies the safety of inventory, whether inventory meets production needs, etc. Hazardous waste belongs to passive inventory; it is a kind of waste material that is not ordered or supplied, but, unlike general waste material, its dangers lead to huge risks to the normal operation of the company if it cannot properly dispose or preserve them. Therefore, the management of hazardous waste inventory in this paper is to study the problem from the perspective of safe production. With the purpose of minimizing safety operation risk, an evaluation index system is constructed according to the influencing factors of production operation risk.

### 1.2. Methodology

As risk assessment is a complex multi-criteria decision-making problem, using MCDM methods can assist decision makers [22]. In this paper, a BWM-TOPSIS hybrid method is employed to evaluate the safety risks associated with hazardous waste inventory. According to BWM, the best and worst criteria are identified first by the decision maker. Pairwise comparisons are then conducted between each of these criteria and the other criteria. A maximin problem is used to determine the weights of different criteria [23]. The TOPSIS method is a multi-criteria decision-making method that ranks the alternatives according to the closeness degrees of alternatives from idea solution [24]. 

Forgionne et al. proposed a gauge as the evaluation index for the quality of journals, and then used the analytic hierarchy process (AHP) method to evaluate the quality of artificial intelligence and decision support system journals [25]. Fogliatto and Albin proposed a hierarchical method based on AHP for calculating the weights or comprehensive performance indicators for products that had quantitative and sensory characteristics [26].Chan and Kumar determined the important decision indicators for global supplier selection, and then proposed an AHP method based on fuzzy extension to resolve the ambiguity problems in the data [27]. Briggs et al. used PROMETHEE I and II methods based on a few strongly conflicting indicators to rank 27 participants [28]. Queiruga et al. used the PROMETHEE method in conjunction with expert surveys to rank Spanish municipalities based on their appropriateness for plant installation [29]. Boran et al. proposed a TOPSIS method combined with intuitionistic fuzzy sets, and, after calculating the intuitionistic fuzzy positive ideal solution and the intuitionistic fuzzy negative ideal solution using Euclidean distance, they obtained the relative proximity coefficients for the alternatives and ranked the alternatives [30]. Awasthi et al. used fuzzy theory to quantify the standard value under uncertain conditions, and then used fuzzy TOPSIS to evaluate and select the best location for the city’s distribution center [31].

Because of the limitations associated with single evaluation methods, multiple methods have often been combined for more accurate multi-criteria decisions. Dagdeviren proposed a comprehensive method using AHP and PROMETHEE for equipment selection, in which AHP was used to analyze the structure of the equipment selection problem and determine the index weight, and the PROMETHEE method was used to obtain the final ranking. It was found that the advantage of PROMETHEE was that there was less contrast and there was no limit when artificially using a standard scale for the evaluation [32]. Kaya and Kahraman used an integrated VIKOR-AHP method to determine the best renewable energy alternatives in Istanbul, in which the selection index weight was determined using an AHP pairwise comparison matrix, with the improved VIKOR used to rank and select the best renewable energy alternative from a set of alternatives and identify compromise solutions for problems with conflicting criteria [33]. Kandakoglu et al. used a SWOT analysis in conjunction with an AHP-TOPSIS method to select a transport registry for the maritime transport industry, for which AHP was used to measure the relative importance of the evaluation criteria for this decision-making body and TOPSIS used to rank the transport registration alternatives [34]. 

Therefore, AHP has been widely used to determine indicator weights through pairwise standard comparisons because it can be easily applied to large-scale, complex decision-making problems involving multiple standards and subjective assessments and can be integrated with other MCDM methods. However, the major challenge with AHP has been its lack of consistency in the pairwise comparison matrix [35]. Rezaei recently proposed BWM and proved that, compared with AHP, BWM requires fewer comparative data and its comparison results are more consistent and therefore more reliable [23]. BWM, proposed in 2015, is rarely used in hazardous waste management, but it is used a lot in decision-making problems. You et al. used a hybrid BWM/TOPSIS method to evaluate the operating performances of grid companies for which BWM was used to determine the indicator weights and TOPSIS was used to rank the grid company performances. Compared to PROMETHEE and VIKOR, TOPSIS has been found to be more suitable when there are many criteria and alternatives, especially for objective or quantitative data, as its ranking process is clearer and easier to implement [36]. Nestico and Somma proposed a comparison between some of the best-known MCDM methods: AHP, ELECTRE, TOPSIS and VIKOR. They pointed out TOPSIS can be applied even in the presence of many criteria and alternatives, and it tends to reject those alternatives that have low values in most of the attributes [37]. As is known, hazardous waste inventory safety risk evaluation in waste-producing companies is a MCDM problem with many criteria and alternatives. Therefore, for calculation simplicity and clarity and because the data used in this study were quantitatively processed, a BWM-TOPSIS method was employed.

Therefore, the purpose of this paper is to develop an effective hazardous waste evaluation system to assist companies reduce the risk of hazardous waste inventory and improve the level of inventory management of hazardous waste.

## 2. Index System Construction

There are several principles that should be followed when selecting evaluation indicators: they should be scientific, typical, universal, quantitative, interdependent, systematic and operational, so that they objectively and comprehensively describe the inventory risks and the corresponding data can easily quantified and collected. Therefore, indicators should be chosen that have scientific significance, are generally accepted and easy to operate and are representative and independent. For practical applications, the selection of a moderate adjustment index should also be considered to cater for the differences in individual companies.

### 2.1. Factor Analysis

The inherent shortcomings in hazardous waste management are the risk factors that affect hazardous waste inventory safety: (1) inventory equipment; (2) management level; (3) nature of the hazardous waste; and (4) operations.

(1) Inventory equipment has a certain useful life. Because of the dangers associated with hazardous waste, the longer the equipment is used, the greater the inventory risk and the higher the need for equipment maintenance. Therefore, both service life and equipment maintenance factors affect inventory safety.
(2) Generally, specialized knowledge, management ability and practical experience can reflect the ability to solve problems in the hazardous waste management. Therefore, management level such as education and professional behavior are also factors that affect inventory safety.
(3) *The National Hazardous Waste List* issued by China in 2016 classified hazardous waste into 50 categories, formulated a *Hazardous Waste Identification Index* to identify unclear hazardous waste and classified the hazardous characteristics: causticity, toxicity, flammability, reactivity and infectivity. Therefore, both the quantity and hazardous waste characteristics are factors that affect inventory safety.
(4) A hazardous waste inventory risk assessment should first consider the operational safety associated with hazardous waste warehousing, storage (packaging and the storage method) and removal, all of which affect inventory safety.

Therefore, from the analysis results for the influencing factors and related literature research, a hazardous waste inventory risk analysis system was established (Table 2).

Because the established risk assessment framework is simple and abstract, and therefore cannot be used to estimate risks in practical applications, the next step was to select the key influencing parameters from these factors and quantify them into indicators based on index selection principles.

### 2.2. Identification and Screening of Indicators

#### 2.2.1. Inventory Equipment

(1) The technical life of inventory equipment is related to its use time; that is, the longer the equipment is used, the greater is the risk. Therefore, to determine the risk, the ratio of the use time of each device to the service life of the device was first calculated, and then the arithmetic average method used to calculate the average number as a criterion, with the larger the ratio, the greater the risk.
(2) Equipment maintenance can be divided into annual maintenance frequency and equipment maintenance timeliness, the criteria for which are:
  - Annual maintenance frequency is the number of times the equipment was quality tested and the operating apparatus checked each year.
  - Equipment maintenance timeliness is the time interval between instrument failure and successful maintenance.

#### 2.2.2. Management Level

(1) The academic qualifications of the inventory staff were divided from low to high from basic education to college education, after which the percentage of college education was used as the criterion, as it was assumed that the higher is the education, the higher is the management level.
(2) Because it is difficult to quantify professional knowledge, management capabilities, or practical experience, this paper defines professionals as persons who have been engaged in hazardous waste management related work for more than three years. The percentage of professionals in the staff was used to measure the professionalism of the inventory management personnel. It was assumed that the higher is the percentage, the higher is the possibility of effective inventory management.

#### 2.2.3. Nature of Hazardous Waste

(1) The greater is the quantity of hazardous waste stored, the higher are the safety requirements for the inventory and the greater is the risk. Therefore, the daily average quantity of hazardous waste was used as the criterion with the measurement unit being the tonne.
(2) The hazardous waste characteristics were classified into five types: corrosive, toxic, flammable, reactive and infectious. The more types of hazardous waste characteristics there are in an inventory, the more difficult it is to manage and the larger the safety risks; therefore, the hazardous waste characteristic types were used as the criteria.

#### 2.2.4. Operations

(1) The primary issue to be considered in the hazardous waste inventory is safety. The hazardous waste inventory packaging should be intact and free of leaks; therefore, the packaging quality was taken as a criterion.
(2) Hazardous waste operations during storage are also a major safety factor, which means that it was necessary to consider the different hazardous waste storage methods and the possibilities of chemical reactions between the hazardous waste. Therefore, the criteria were:
  - Storage method: The selection of different storage methods for different types of hazardous wastes.
  - Separation operation: The separation of hazardous waste that has a risk of chemical reactions.

Therefore, four criteria and ten sub-criteria were finally selected, as shown in Table 3.

As the operation-related criteria are difficult to quantify, this paper uses an expert scoring method to design a questionnaire scale to convert the three operation category influencing factors into three questions. This paper is based on the industrial resources of the National Hazardous Waste Disposal Engineering Technology Center. Ten professionals in this center were interviewed and invited to form an expert group. On a scale of 1–5, the arithmetic average of the data obtained from each question was used as the indicator.

## 3. BWM and TOPSIS for Risk Evaluation

**Figure 1:**
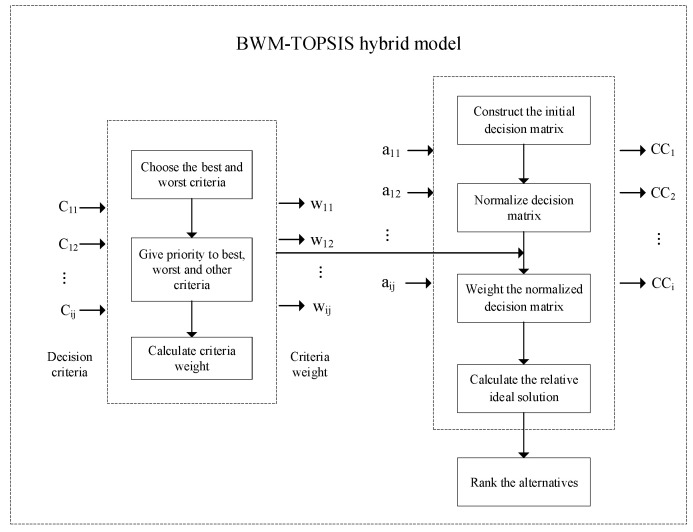
Hazardous waste inventory risk evaluation methodological framework.

Because it has fewer pairwise comparisons and better consistency, this paper uses BWM to calculate the evaluation index weights, and then the TOPSIS method in combination with the BWM weight determination is used to calculate the distance from the alternative to the ideal solution and rank the alternatives. The methodological framework is shown in Figure 1.

### 3.1. BWM Calculation of Criteria Weights 

The evaluation index weight needs to be determined first for the hazardous waste inventory safety evaluation. It is very important that the criteria weight determination be scientific and reasonable as it significantly affects the comprehensive hazardous waste inventory safety risk evaluation and the associated management decisions. Therefore, a new multi-indicator decision-making method, BWM, was employed to determine the criteria weight as it has been proven to be superior to AHP [23,46,47].

The main steps in this method were as proposed by Rezaei [23]:

Step 1: Determine the decision criteria.

Adopt the decision index system constructed in the Section 3 and express it as n criteria: {c_1_, c_2_, …, c_n_}.

Step 2: Determine the best criterion c_B_ asnd the worst criterion c_W_ from the criteria and sub-criteria.

Step 3: Give priority to the best criterion relative to the other criteria on a scale of 1–9. A scale of 1–9 determines preferences for best criterion over all other criteria. The vector for the best criterion over the other criteria is expressed as:

AB = (aB1, aB2, ……, aBn)(1)

where a_Bj_ represents the priority of the best criterion c_B_ over any other criteria c_j_. In this case, a_BB_ = 1.

Step 4: Similarly, using a scale of 1–9, determine the priority of all other criteria over the worst criterion. The vector for the other criteria that are better than the worst criterion can be expressed as:

A_W_ = (a_1W_, a_2W_, ……, a_nW_)^T^ (2)

where a_jW_ represents the priority of any other criteria c_j_ over the worst criterion c_W_. In this case, a_WW_ = 1.

Step 5: Optimize all criteria weights, ($w_{1}^{*}$, $w_{2}^{*}$, ……, $w_{3}^{*}$).

The purpose is to calculate the index weight to minimize the maximum absolute difference of all j in the following set {|w_B_-a_Bj_w_j_|, |w_j_-a_jW_w_W_|}. The following minimax models were then established:

min max {|w_B_-a_Bj_w_j_|, |w_j_-a_jW_w_W_|} s.t.∑_j_w_j_ = 1 w_j_ ≥ 0, for all j (3)

Model (1), as discussed above, can be solved as a linear model as follows:

(4) $$\min\xi \;\mathrm{s.t.}\;|\frac{w_{B}}{w_{j}}-a_{\mathrm{Bj}}|\leq \xi ,\;\mathrm{for}\;\mathrm{all}\;j\;|\frac{w_{j}}{w_{w}}-a_{\mathrm{jW}}|\leq \xi ,\mathrm{for}\;\mathrm{all}\;j\;\sum_{j}w_{j}=1\;w_{j}\geq 0,\mathrm{for}\;\mathrm{all}$$

The calculation of Model (2) obtained the optimal weights ($w_{1}^{*}$, $w_{2}^{*}$, ……, $w_{n}^{*}$) and the optimal value ξ^*^.

Step 6: Calculate the consistency ratio. The closer the ratio is to 0, the higher is the consistency.

For different a_BW_∈{1, 2, ……,9}, different maximum possible ξ (maxξ) can be calculated, which are then used in the consistency index table (Table 4).

Use ξ^*^ and the corresponding consistency index to calculate the consistency ratio:

(5) $$\mathrm{Consistency}\;\mathrm{Ratio}=\frac{\xi^{*}}{Consistency\;Index}$$

### 3.2. TOPSIS for Ranking the Alternatives

The TOPSIS method is a multi-criteria decision-making method that ranks alternatives based on how close the alternatives are to the ideal, with the ideal being the hypothesized best alternative. Suppose there are m alternatives A = {A_1_, A_2_, ……, A_m_} and n criteria C = {C_1_, C_2_, ……, C_n_}, the specific steps for the TOPSIS [24,48] are:

Step 1: Establish the initial decision matrix A.

The original data for the alternative criteria are subjected to vector standardization processing (such as the inverse method to convert the low-optimal criteria into high-optimal criteria) to obtain the initial decision matrix A as follows:

(6) $$A={\left(a_{\mathrm{ij}}\right)}_{m\times n}=\left(\begin{array}{ccc} a_{11} & \cdots & a_{1n}\\ \vdots & \ddots & \vdots \\ a_{m1} & \cdots & a_{mn} \end{array}\right)$$

The larger are the values of some criteria, the better, which are the high-optimal criteria, and the smaller are the value of some criteria, the better, which are the low-optimal criteria. To unify and facilitate the calculations, the low-optimal criteria must be standardized into high-optimal criteria.

a_ij_ is the value of the jth criterion for the ith alternative; i = 1, 2, ……, m, and j = 1, 2, ……, n.

Step 2: Standardize the initial decision matrix A to get the normalized decision matrix B.

As different criteria have different units and quantities, to avoid numerical differences and ensure mathematical compatibility, the initial decision matrix needs to be standardized:

(7) $$b_{\mathrm{ij}}=a_{\mathrm{ij}}/\sqrt{\sum a_{\mathrm{ij}}^{2}}$$

Each value in the initial decision matrix is divided by the square root of the corresponding sum of squares in each column of data. a_ij_ is the value of the jth index of the ith alternative and b_ij_ is the normalized value of the jth index of the ith alternative.

After normalization, the normalized decision matrix B is obtained.

(8) $$B={\left(b_{\mathrm{ij}}\right)}_{m\times n}=\left(\begin{array}{ccc} b_{11} & \cdots & b_{1n}\\ \vdots & \ddots & \vdots \\ b_{m1} & \cdots & b_{mn} \end{array}\right)$$

Step 3: Construct a weighted normalized decision matrix C.

The weight for the normalized decision matrix C is obtained by multiplying the values of the index weights determined in the first stage by the values of each column of the corresponding normalized decision matrix:

(9) $$C={\left(c_{\mathrm{ij}}\right)}_{m\times n}=\left(\begin{array}{ccc} w_{1}\times b_{11} & \cdots & w_{n}\times b_{1n}\\ \vdots & \ddots & \vdots \\ w_{1}\times b_{m1} & \cdots & w_{n}\times b_{mn} \end{array}\right)$$

where c_ij_ represents the weighted normalized value for the ith index of the jth alternative.

Step 4: Determine the positive ideal set C^+^ and the negative ideal set C^−^ according to the weighted normalized decision matrix C.

The maximum and minimum values in each column of matrix C are selected. All the maximum values constitute the positive ideal set C^+^ and all the minimum values constitute the negative ideal set C^−^.

(10) $$\{\begin{array}{c} C^{+}=\left(c_{j}^{+}\right)=\left\{\underset{i}{\max}c_{ij}|j=1,2,\ldots \ldots ,n\right\}\\ C^{-}=\left(c_{j}^{-}\right)=\left\{\underset{i}{\min}c_{ij}|j=1,2,\ldots \ldots ,n\right\} \end{array}$$

Step 5: Calculate the distance from each alternative to the positive and negative ideal solutions.

This paper uses Euclidean distance to calculate the distance from the alternative to the positive and negative ideal solutions:

(11) $$\{\begin{array}{c} D_{i}^{+}=\sqrt{\overset{n}{\underset{j=1}{\sum }}{\left(c_{\mathrm{ij}}-c_{j}^{+}\right)}^{2}}\\ D_{i}^{-}=\sqrt{\overset{n}{\underset{j=1}{\sum }}{\left(c_{\mathrm{ij}}-c_{j}^{-}\right)}^{2}} \end{array}$$

where $D_{i}^{+}$ represents the distance from alternative i to the positive ideal solution, and $D_{i}^{-}$ represents the distance from alternative i to the negative ideal solution.

Step 6: Calculate the relative ideal solution.

The relative ideal solution is the value closest to the positive ideal solution and farthest from the negative ideal solution:

(12) $${\mathrm{CC}}_{i}=\frac{D_{i}^{-}}{D_{i}^{+}+D_{i}^{-}}$$

CC_i_ represents the relative ideal solution for the ith alternative.

Step 7: Rank the alternatives

The alternatives are ranked based on the CC_i_ values, with the alternative having a maximum or minimum CC_i_ being the optimal solution. 

## 4. Case Analysis

The reliability of the developed safety hazardous waste inventory risk evaluation model was verified using an actual case.

### 4.1. Case Enterprise

Four chemical companies in Sichuan Province, China, were chosen to test the hazardous waste inventory safety risk evaluation; the details are not given because the information involves the privacy of the companies. Each company generated large quantities of hazardous waste every day and are located in rapidly developing areas that have weak environmental management. Therefore, it is possible to apply the results in developing countries. 

### 4.2. Calculating the Criteria Weights Using BWM

After determining the criteria, the steps described in Section 3.1 were followed to calculate the criteria weights. As the criteria in this study were divided into primary criteria and sub-criteria, the primary criteria weights were first calculated, followed by the sub-criteria and, finally, the total weight.

Using the Delphi method, 10 experts determined the best criteria and worst criteria individually based on their professional experience. Then, based on the consensus process, the expert group determined that operations (c_4_) and equipment (c_1_) in the primary criteria were the best criteria and worst criteria, respectively. Then, giving their priorities on the other criteria in a similar way, the priorities of the best criterion and worst criterion relative to the other criteria on a scale of 1–9 was determined:

A(P)_B_ = (9, 5, 3, 1); A(P)_W_ = (1, 2, 3, 9) (13)

By substituting the data in the above priorities into Equation (4), Equation (14) was derived:

(14) $$\min\xi \;\mathrm{s.t.}\;|\frac{w_{4}}{w_{1}}-9|\leq \xi ,\;|\frac{w_{4}}{w_{2}}-5|\leq \xi ,\;|\frac{w_{4}}{w_{3}}-3|\leq \xi ,\;|\frac{w_{2}}{w_{1}}-2|\leq \xi ,\;|\frac{w_{3}}{w_{1}}-3|\leq \xi ,\;w_{1}+w_{2}+w_{3}+w_{4}=1\;w_{1},w_{2},w_{3},w_{4}\geq 0.$$

Equation (14) was calculated using LINGO, from which w_1_ = 0.067, w_2_ = 0.125, w_3_ = 0.200, w_4_ = 0.608 and ξ = 0.127 were obtained. Depending on the value for ξ, Equation (3) was used to calculate the consistency ratio, which was CR = 0.024, indicating that the consistency was very high. 

The primary equipment (c_1_) and operations (c_4_) criteria each had three sub-criteria. Similar to the above, using the expert group opinions, the priorities were obtained:

A(E)_B_ = (1, 2, 8); A(E)_W_ = (8, 4, 1) (15)

A(O)_B_ = (1, 2, 7); A(O)_W_ = (7, 3, 1) (16)

The data were substituted into Equation (4) and calculated using LINGO:

w_11_ = 0.615, w_12_ = 0.308, w_13_ = 0.077 (17)

w_41_ = 0.622, w_42_ = 0.287, w_43_ = 0.091 (18)

As there were only two sub-criteria on the management level (c_2_) and the nature of hazardous waste level (c_3_) in the primary criteria, the weights were directly determined based on the sub-criteria priorities determined by the expert group:

w_21_ = 0.333, w_22_ = 0.667, w_31_ = 0.750, w_32_ = 0.250 (19)

The total weight of the evaluation criteria was then determined based on the weight of the primary criteria and the primary criteria sub-criteria, as shown in Table 5.

### 4.3. Ranking the Case Companies Using TOPSIS

After calculating the criteria weights, the next step was to rank the case companies using the steps described in Section 3.2. The data for these four case companies were obtained from on-site surveys and the questionnaire scale shown in Table 6.

The initial criteria data were processed using vector standardization and the low-optimal criteria were converted into high-optimal criteria.

(20) $$A={\left(a_{\mathrm{ij}}\right)}_{4\times 10}=\left(\begin{array}{cc} 0.357 & \begin{array}{ccc} 10 & 1.000 & \begin{array}{ccc} 0.250 & 0.167 & \begin{array}{ccc} 0.278 & 0.333 & \begin{array}{ccc} 3.8 & 4.2 & 2.4 \end{array} \end{array} \end{array} \end{array}\\ \begin{array}{c} 0.486\\ 0.888\\ 0.673 \end{array} & \begin{array}{ccc} 5 & 0.143 & \begin{array}{ccc} 0.333 & 0.286 & \begin{array}{ccc} 0.175 & 0.250 & \begin{array}{ccc} 4.2 & 4.0 & 2.0 \end{array} \end{array} \end{array}\\ 1 & 0.143 & \begin{array}{ccc} 0.429 & 0.140 & \begin{array}{ccc} 0.233 & 0.250 & \begin{array}{ccc} 3.8 & 3.8 & 2.8 \end{array} \end{array} \end{array}\\ 12 & 0.333 & \begin{array}{ccc} 0.714 & 0.238 & \begin{array}{ccc} 0.122 & 0.200 & \begin{array}{ccc} 4.6 & 2.8 & 1.6 \end{array} \end{array} \end{array} \end{array} \end{array}\right)$$

Equation (7) was used for the standardization to obtain the normalized decision matrix B:

(21) $$B={\left(b_{\mathrm{ij}}\right)}_{4\times 10}=\left(\begin{array}{cc} 0.282 & \begin{array}{ccc} 0.609 & 0.932 & \begin{array}{ccc} 0.268 & 0.387 & \begin{array}{ccc} 0.661 & 0.634 & \begin{array}{ccc} 0.462 & 0.562 & 0.535 \end{array} \end{array} \end{array} \end{array}\\ \begin{array}{c} 0.384\\ 0.701\\ 0.531 \end{array} & \begin{array}{ccc} 0.304 & 0.133 & \begin{array}{ccc} 0.358 & 0.663 & \begin{array}{ccc} 0.416 & 0.476 & \begin{array}{ccc} 0.511 & 0.535 & 0.445 \end{array} \end{array} \end{array}\\ 0.061 & 0.133 & \begin{array}{ccc} 0.461 & 0.325 & \begin{array}{ccc} 0.554 & 0.476 & \begin{array}{ccc} 0.462 & 0.508 & 0.624 \end{array} \end{array} \end{array}\\ 0.730 & 0.310 & \begin{array}{ccc} 0.767 & 0.552 & \begin{array}{ccc} 0.290 & 0.381 & \begin{array}{ccc} 0.559 & 0.374 & 0.356 \end{array} \end{array} \end{array} \end{array} \end{array}\right)$$

The weights calculated in Section 4.2 were substituted into Equation (9) to construct the normalized weighted decision matrix C:

(22) $$C={\left(c_{\mathrm{ij}}\right)}_{4\times 10}=\left(\begin{array}{cc} 0.012 & \begin{array}{ccc} 0.013 & 0.005 & \begin{array}{ccc} 0.011 & 0.032 & \begin{array}{ccc} 0.099 & 0.032 & \begin{array}{ccc} 0.175 & 0.098 & 0.029 \end{array} \end{array} \end{array} \end{array}\\ \begin{array}{c} 0.016\\ 0.029\\ 0.022 \end{array} & \begin{array}{ccc} 0.006 & 0.001 & \begin{array}{ccc} 0.015 & 0.055 & \begin{array}{ccc} 0.062 & 0.024 & \begin{array}{ccc} 0.193 & 0.093 & 0.024 \end{array} \end{array} \end{array}\\ 0.001 & 0.001 & \begin{array}{ccc} 0.019 & 0.027 & \begin{array}{ccc} 0.083 & 0.024 & \begin{array}{ccc} 0.175 & 0.088 & 0.034 \end{array} \end{array} \end{array}\\ 0.015 & 0.002 & \begin{array}{ccc} 0.032 & 0.046 & \begin{array}{ccc} 0.043 & 0.019 & \begin{array}{ccc} 0.211 & 0.065 & 0.020 \end{array} \end{array} \end{array} \end{array} \end{array}\right)$$

From Equation (10), the positive ideal set c^+^ and the negative ideal set c^−^ for matrix C were obtained.

(23) $$\{\begin{array}{c} c^{+}=\left(0.029,\;0.015,\;0.005,\;0.032,\;0.055,\;0.099,\;0.032,\;0.211,\;0.098,\;0.034\right)\\ c^{-}=\left(0.012,\;0.001,\;0.001,\;0.011,\;0.027,\;0.043,\;0.019,\;0.175,\;0.065,\;0.020\right) \end{array}$$

From Equation (11):

(24) $$\{\begin{array}{c} D^{+}=\left(0.051,\;0.049,\;0.054,\;0.068\right)\\ D^{-}=\left(0.068,\;0.049,\;0.052,\;0.049\right) \end{array}$$

The relative ideal solution from equation (12) was calculated: CC = (0.572, 0.498, 0.493, 0.419): The final results indicate: CC_1_ > CC_2_ > CC_3_ > CC_4_.

Therefore, the hazardous waste inventory safety risk in Case Company 1 was the smallest, followed by Case Company 2, Case Company 3 and Case Company 4.

### 4.4. Discussion

From the criteria weights in Table 5, it can be seen that the weight for operations (c_4_) was 0.608 at its maximum, followed by the nature of the hazardous waste (c_3_). Therefore, based on the primary criteria, to reduce the hazardous waste inventory risk, the operations need to be improved first, with the key deciding factor being the packaging quality (c_41_); that is, the most important strategy is to improve the hazardous waste packaging quality.

From the initial case companies’ data in Table 6, Case Company 4 has the maximum 4.6 in the high-optimal criteria packaging quality (c_41_), but it has the greatest hazardous waste inventory safety risk of the four case companies. This is because, in the high-optimal criteria packaging quality (c_41_), the values of the four case companies are 3.8, 4.2, 3.8, and 4.6 in order, with a small gap; however, in the high-optimal criteria storage method (c_42_), Case Company 4 has the minimum 2.8, especially in the low-optimal criteria quantity (c_31_) Case Company 4 has the maximum 8.2, which is more than double that of Case Company 1. Therefore, for Case Company 4, the inventory risk should be reduced by improving storage method and the amount of hazardous waste.

This case verified the scientificity, rationality and convenience of the developed hazardous waste inventory safety risk evaluation system and gives guidance to companies for the assessment of their own hazardous waste inventory risk. 

## 5. Conclusions

The recent increase in hazardous waste has not received enough attention from industry management. Therefore, because of the inventory risks associated with hazardous waste, this paper develops a hazardous waste inventory risk evaluation index system from the four aspects of equipment, management level, nature of hazardous waste and operations. A mixed multi-criteria decision-making method, BWM-TOPSIS, was employed to solve the risk evaluation problems associated with hazardous waste inventory, proving the rationality of the proposed model in four practical cases. The results show that Case Company 4 has the greatest risk of hazardous waste inventory, which should be reduced by improving storage method and the amount of hazardous waste.

Therefore, this paper proposes a method for the management of hazardous waste inventory of waste-producing companies, expands the scope of inventory safety management and strengthens the source management of hazardous waste based on previous research. This paper has the following innovations.

(1) A novel risk evaluation index system for hazardous waste inventory was constructed to assist companies reduce the risk of hazardous waste inventory and improve the level of inventory management of hazardous waste.
(2) For the first time, this study used the BWM-TOPSIS model to evaluate the inventory risk and develop a new decision-making framework for hazardous waste inventory risk evaluation.
(3) A new inventory management method was proposed from a safe production perspective to improve the scientificity of hazardous waste inventory management, reduce the risk of hazardous waste inventory management and provide a scientific management basis for decision makers.

However, there were some limitations and the following research directions could improve the accuracy and viability of the proposed model.

(1) This paper selects equipment, management level, nature of hazardous waste and operations to establish the risk evaluation criteria. However, these criteria were all on-site factors and no off-site factors were considered, such as effects on nearby residents and nature reserves. Therefore, future research could consider adding off-site factors to the inventory risk evaluation model. In this assumption, considering the damage of hazardous waste to the surrounding environment, the warehouse location of the waste-producing companies may become a key factor affecting the evaluation results.
(2) The hazardous waste inventory risk evaluation model used an expert scoring method to quantify the operational criteria, which could be considered somewhat subjective. Therefore, the next step could be to consider adding fuzzy theory to increase the objectivity of the model. The limit of this hypothesis is that experts need to understand fuzzy theory, and this limit can be overcome by introducing language variables to allow experts to directly choose qualitative data.

## Figures and Tables

**Table 1:** The 2010–2017 international hazardous waste output from the United Nations Statistics Division.

Country	2010	2011	2012	2013	2014	2015	2016	2017
Armenia	435,398	462,896	470,506	579,050	576,419	555,077	615,471	543,232
Austria	1,472,864	…	1,065,888	…	1,272,288	…	1,260,953	…
Belarus	918,200	943,196	1,322,792	1,415,421	1,723,975	1,207,798	1,626,610	1,668,060
China	15,867,550	34,312,200	34,652,400	31,568,900	36,335,200	39,761,100	53,473,000	69,368,896
Denmark	1,224,795	…	1,216,905	…	1,715,907	…	2,010,740	…
Germany	19,931,452	…	21,983,896	…	21,812,660	…	23,039,154	…
Iceland	8,304	…	16,263	…	36,250	…	47,857	…
Italy	8,543,415	…	8,987,032	…	8,877,164	…	9,706,964	…
Kyrgyzstan	5,806,792	10,152,943	4,930,216	7,957,260	10,223,015	10,498,943	12,377,486	12,648,247
Poland	1,491,845	…	1,737,024	…	1,681,037	…	1,917,134	…
Singapore	434,000	432,600	280,500	332,800	411,180	446,870	478,990	471,450

**Table 2:** Hazardous waste inventory risk factor analysis system.

First-Level Influencing Factors	Secondary Influencing Factors	Related Literature
Equipment	Equipment usage time	Li et al. established an evaluation index system for groundwater pollution near hazardous waste landfills, which had landfill management field equipment maintenance as an influencing factor, which had two indicators: routine maintenance frequency and the equipment repair and replacement timeliness [38]. Schroer and Modarres considered the same equipment components in their multi-unit nuclear power plant probabilistic risk assessment and pointed out that the design, installation and maintenance of these components were almost the same, which made them susceptible to traditional common cause failures [39]. Hsu et al. studied the operational safety risk factors for dangerous goods air cargo and included the lack of equipment in the customs operations as a risk factor [40].
	Equipment maintenance	
Management level	Education	Li et al. included landfill management employee competence as an important factor affecting groundwater when establishing groundwater pollution risk assessment indicators and used the percentage of professionals in the staff to measure the human impact of daily landfill operations [38]. Hsu et al. included the lack of qualified and experienced inspectors in customs operations as a risk factor for air cargo dangerous goods [40]. Gumus used an improved Delphi method to identify eight evaluation criteria for hazardous waste shipping, which included the ability to solve problems [41]. Trucco et al. integrated human factors and organizational factors into a risk analysis for marine transportation and pointed out that passenger and crew behaviors were affected by management and organizational structures [42].
	Professional	
The nature of hazardous waste	Quantity	Li et al. included the nature of waste filtrate as an influencing factor in groundwater pollution risk in a groundwater pollution risk assessment index near hazardous waste landfills, with the most important of these being the chemical composition of the leachate [38]. When developing a quantitative railway station risk assessment, Glickman and Erkut included the risks associated with the receipt and storage of dangerous goods tankers, which in this case involved six different chemicals. For each dangerous substance, an average monthly quantity and the hazards that required the most attention were specified [43]. Escher et al. correlated drug toxicity data with predicted concentrations in different wastewater streams when developing an environmental toxicology and risk assessment for drugs in hospital wastewater to assess the overall potential risks in different situations [44].
	Hazardous characteristics	
Operation	Pack	Schroer and Modarres examined human dependence in a probabilistic risk assessment for a multi-unit nuclear power plant, finding that the lack of monitoring, maintenance and cleaning operations could increase the potential risks [39]. Hsu et al. considered operations such as insufficient packaging and the failure to regulate cargo unloading as dangerous factors in the risk assessment of air cargo dangerous goods [40]. Ho and Chen counted the list of laboratory accidents that had occurred at Taiwan University since 2000 and prioritized 10 potential risk factors based on the number of risk priorities: the use of funnels or drain pans for waste liquid collection, decentralized storage, container materials and other operational factors [45].

**Table 3:** Hazardous waste inventory safety risk assessment index system.

Criteria	Sub-Criteria	Definition
Equipment $\left(c_{1}\right)$	Equipment usage time $\left(c_{11}\right)$	Equipment usage time percentage
	Annual maintenance frequency $\left(c_{12}\right)$	number of times the equipment is quality tested and operating apparatus checked each year
	Timeliness of maintenance $\left(c_{13}\right)$	the time interval between instrument failure and successful maintenance.
Management level ($c_{2})$	College education percentage $\left(c_{21}\right)$	University graduates as a percentage of all warehouse management staff
	Professional percentage $\left(c_{22}\right)$	Proportion of risk management related professionals in all warehouse management staff
Nature of hazardous waste $\left(c_{3}\right)$	Quantity $\left(c_{31}\right)$	Daily average hazardous waste quantity
	Hazardous waste characteristics $\left(c_{32}\right)$	Hazardous waste characteristics: corrosive, toxic, flammable, reactive and infectious
Operation $\left(c_{4}\right)$	Packing quality $\left(c_{41}\right)$	Packaging must be intact
	Storage method $\left(c_{42}\right)$	Different storage methods for different types of hazardous waste
	Separation operation ($c_{43})$	Separation of hazardous waste that has potential chemical reactions

**Table 4:** Consistency index table.

a_BW_	1	2	3	4	5	6	7	8	9
Consistency Index (Max ξ)	0.00	0.44	1.00	1.63	2.30	3.00	3.73	4.47	5.23

**Table 5:** Risk evaluation criteria weights.

Primary Criteria	Primary Criteria Weight	Sub-Criteria	Sub-Criteria Weight	Total Weight	Ranking
		Equipment usage time	0.615	0.041	8
Equipment	0.067	Annual maintenance frequency	0.308	0.021	9
		Maintenance timeliness	0.077	0.005	10
Management	0.125	College education percentage	0.333	0.042	7
level		Professional percentage	0.667	0.083	4
Nature of hazardous	0.200	Quantity	0.750	0.150	3
Waste		Hazardous waste characteristics	0.250	0.050	6
		Packing quality	0.622	0.378	1
Operation	0.608	Storage method	0.287	0.174	2
		Separation operation	0.091	0.055	5

**Table 6:** Initial case companies’ data.

	Enterprise 1	Enterprise 2	Enterprise 3	Enterprise 4
Equipment usage time	0.643	0.514	0.112	0.327
Annual maintenance frequency	10	5	1	12
Maintenance timeliness	1	7	7	3
College education percentage	0.25	0.333	0.429	0.714
Professional percentage	0.167	0.286	0.14	0.238
Quantity	3.6	5.7	4.3	8.2
Hazardous waste characteristics	3	4	4	5
Packing quality	3.8	4.2	3.8	4.6
Storage method	4.2	4	3.8	2.8
Separation operation	2.4	2	2.8	1.6
